# IMU-Based Online Kinematic Calibration of Robot Manipulator

**DOI:** 10.1155/2013/139738

**Published:** 2013-11-05

**Authors:** Guanglong Du, Ping Zhang

**Affiliations:** School of Computer Science & Engineering, South China University of Technology, Guangzhou 510006, China

## Abstract

Robot calibration is a useful diagnostic method for improving the positioning accuracy in robot production and maintenance. An online robot self-calibration method based on inertial measurement unit (IMU) is presented in this paper. The method requires that the IMU is rigidly attached to the robot manipulator, which makes it possible to obtain the orientation of the manipulator with the orientation of the IMU in real time. This paper proposed an efficient approach which incorporates Factored Quaternion Algorithm (FQA) and Kalman Filter (KF) to estimate the orientation of the IMU. Then, an Extended Kalman Filter (EKF) is used to estimate kinematic parameter errors. Using this proposed orientation estimation method will result in improved reliability and accuracy in determining the orientation of the manipulator. Compared with the existing vision-based self-calibration methods, the great advantage of this method is that it does not need the complex steps, such as camera calibration, images capture, and corner detection, which make the robot calibration procedure more autonomous in a dynamic manufacturing environment. Experimental studies on a GOOGOL GRB3016 robot show that this method has better accuracy, convenience, and effectiveness than vision-based methods.

## 1. Introduction

Because of the manufacturing and assembly tolerance, the actual kinematic parameters of a robot deviate from their nominal values, which are referred to as kinematic errors. The kinematic errors would result in the errors of the robot tool if the nominal kinematics were used to estimate the pose of the robot. With the restriction of cost, the kinematic calibration is an effective way to improve the absolute accuracy of robots. Nowadays, calibration tasks use a lot of measurement techniques like coordinate measuring machines, laser tracking interferometer systems, and inexpensive customized fixtures [1, 2]. These systems are not only very expensive but also not friendly to use or with low working volume. A system which is used in a dynamic environment is expected to perform calibration without any external expensive calibration apparatus and elaborate setups, which means self-calibration.

Self-calibration techniques can be classified into two kinds: (1) redundant sensor approach and (2) motion constraint approach.

To increase the degrees-of-sensing over DOF, the redundant sensors approach includes one or more redundant rotary sensors to the proper passive joints of the manipulator. There is a self-calibration method for parallel mechanisms with a case study on Stewart platform which is proposed by Zhuang in [3]. He used forward and inverse kinematics with six rotary encoders for three objective functions of parameter identification. Khalil and Besnard [4] installed two orthogonally allocated inclinometers to the tool to calibrate the Stewart platform except the redundant sensors which are mentioned above. However, there are some limitations of these methods. One of them is that some kinematic parameters orthogonally are not independent of the error models and the position and/or orientation of the tool on the platform cannot be calibrated.

For the other approach, that is the motion constraint approach, the mobility of the resultant system will be lower than its inherent degrees-of-sensing by fixing one or more passive joints or constraining partial DOF of the manipulator so that the calibration algorithm can be performed [5]. Bennett and Hollerbach [6] lowered the mobility of the tool of a serial manipulator and performed self-calibration using only the inherent joint sensors in the manipulator. And this idea was used and extended to calibrate a robot system with a hand-mounted instrumented stereo camera [7]. However, the position and/or orientation of the tool on the platform cannot be calibrated, and some parameter errors related to the locked passive joints may become unobservable in the calibration algorithm because of the mobility constraints.

To solve these limitations, advances in robot calibration allow the researchers to use a hand-mounted camera to calibrate a robot instead of using measurements from passive joints or imposing mechanical constraints. Compared with those mechanical measuring devices, this camera system costs less and it is easier to use and more accurate. The traditional vision-based methods [8–15] to calibrate a robot require the precise 3D fixtures measured in a reference coordinate system and the procedure is inconvenient and time consuming and it may not be feasible for some applications. The self-calibration methods [16, 17] assume that the camera is rigidly attached to the robot tool. Closed-loop methods “virtual closed kinematic chain” proposed in [18–20], use the joint angle measurements already in the robot and can be considered self-calibrating. A method uses laser to capture robot position data to model the stiffness of the manipulator [21] and predict kinematic parameters [22–26]. O'Brian et al. [27] used a magnetic motion to capture robot data to estimate the kinematic parameters. Renaud et al. [28] and Rauf et al. [29] used a vision-based measuring device and a pose measurement device for kinematic calibration, respectively. Santolaria et al. [30] employed a continuous data capture method by using a ball bar gauge and a coupling probe to estimate the kinematic parameters. However, these approaches have a limitation; that is, the calibration is completed off-line. The optimization technique was based on the measured positions of the EE. The parameter error was minimized in the measured positions, but the error increased in very different positions. Moreover, the parameter error increased while the robot withstood different loads. When the robot is used in high-temperature or high-pressure environments, such as deep sea or outer space, the shapes of the robot links are easy to change. Therefore, online calibration is an indispensable method to rectify the kinematic parameters in real time. 

**Figure 1:**
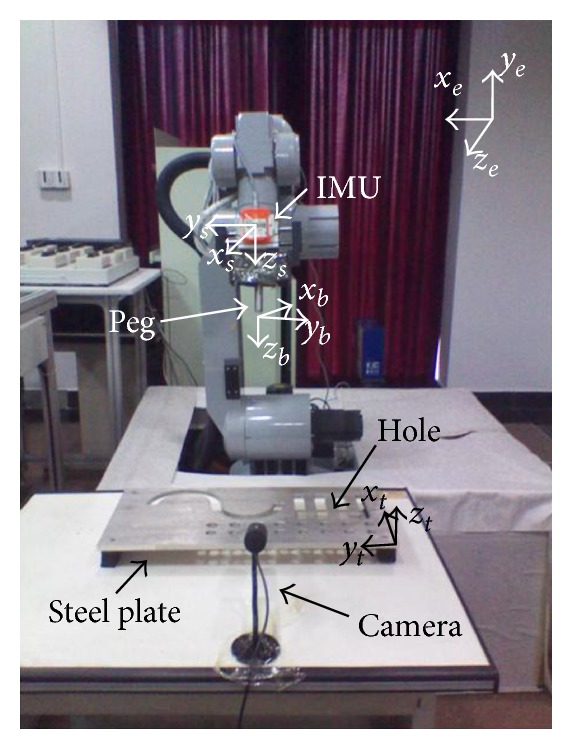
Structure of the system.

In this paper, we propose an original approach of online robot calibration using IMU to measure the robot poses. In our method, an IMU is required to rigidly attach to the robot tool (Figure 1) to measure the robot pose in real time. In order to reduce the effect of the noise and improve the accuracy, we proposed a method combined FQA and KF to estimate the orientation of the IMU. Finally, an EKF is used to estimate differential errors of individual kinematic parameters. Unlike existing vision-based self-calibration methods, the described method does not require special complex steps such as camera calibration and corner detection. Moreover, this method does not require robot to make the motion for capturing the images, which makes our method more efficient.

The remainder of the paper is organized as follows.  Section 2 provides kinematic modeling for the serial robot. In Section 3, a method of pose measurement using IMU is presented. Parameters identification algorithm is proposed in Section 4. In Section 5, an EKF is detailed to estimate the kinematic parameter errors. Finally, the experimental results are shown in Section 6 and we conclude the paper in Section 7.

## 2. Kinematic Modeling 

A robot kinematic model relates the robot joint coordinate to the pose of the robot tool. A robot kinematic model should meet the following rules for the kinematic parameter identification [21–23].

1. Completeness: the robot kinematic model should have enough parameters to define any possible deviation from the nominal values [24].
2. Continuity: any small changes in the structure of the robot must correspond to small changes in kinematic parameters [21].
3. Minimality: the kinematic model must include only a minimal number of parameters [3].

**Figure 2:**
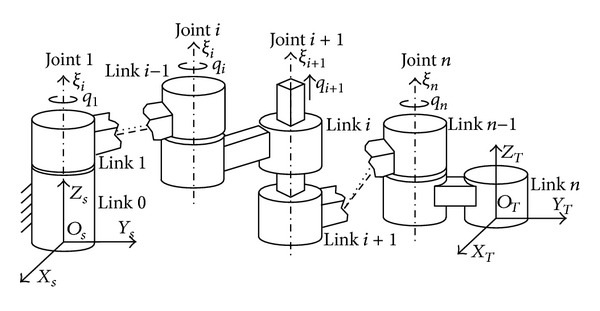
Forward kinematics of an *n*-DOF robot.

Many researchers have found suitable kinematic models for robot since 1980s, such as Hayati et al. models [25–27], Veitschegger and Wu's model [28], Stone and Sanderson's S-model [29], and Zhuang et al. model [30]. The standard Denavit-Hartenberg (DH) [31] convention is the most often used to describe the robot kinematics (Figure 2). The error models of DH are not continuous for robots that possess parallel joint axes. To avoid the singularity of DH convention, the DH modeling or Hayati modeling convention were used, respectively. The singularity-free calibration model prevents the use of a single minimal modeling convention which can be used to identify all possible robot parameters.

The robot tool position and orientation are defined according to the controller conventions. Through the consecutive homogeneous transformations from the base coordinate to the robot tool coordinate (Figure 2), the kinematic equation can be defined as

(1) TN0=TN0(v)=T10T21⋯TNN−1=∏i=1NTii−1,

where *T*_*i*_^*i*−1^ is the translation matrix from *i* − 1 coordinate to *i* coordinate, *N* is the number of joints (or joints coordinates), **v** = [*v*_1_^*T*^ *v*_2_^*T*^ ⋯ *v*_*N*_^*T*^]^*T*^ is the parameter vector for the robot, and *v*_*i*_^*T*^ is the link parameter vector, which includes the joint errors

(2) $$\begin{array}{c} v_{i}=v_{i}^{\prime }+\Delta v_{i}, \end{array}$$

where *v*_*i*_′ is the nominal vector for the joint *i* and Δ*v* = [Δ*v*_1_^*T*^Δ*v*_2_^*T*^ ⋯ Δ*v*_*N*_^*T*^]^*T*^ is the link parameter error vector for the joint *i*. The exact kinematic equation is

(3) KN0=KN0(v)=K10K21⋯KNN−1=∏i=1NKii−1,

(4) Kii−1=Tii−1(vi′+Δvi)=Tii+ΔTi.

Taking the joint variables into consideration, thus,

(5) KN0=TN0+ΔT,  ΔT=ΔT(u,Δv),

where **u** = [*θ*_1_^*T*^ *θ*_2_^*T*^ ⋯ *θ*_*N*_^*T*^]^*T*^ is a vector of joint variables. Thus,  *K*_*N*_^0^ : *ℜ* ^*n*^ × *ℜ* ^*N*^ is a function of **u** and Δ*v*.

## 3. Pose Measurement Using IMU

### 3.1. Factored Quaternion Algorithm (FQA)

 The FQA presented in [32], which is based on Earth gravity and magnetic field measurements, is for estimating the orientation of a rigid body. This algorithm is only applied for the static or slow-moving rigid body. In order to be applicable to situations in which relatively large linear accelerations occur, we use* KF* fusion algorithm together with angular rate information to estimate the orientation of (slow-moving or fast-moving) dynamic body in the next section.

**Figure 3:**
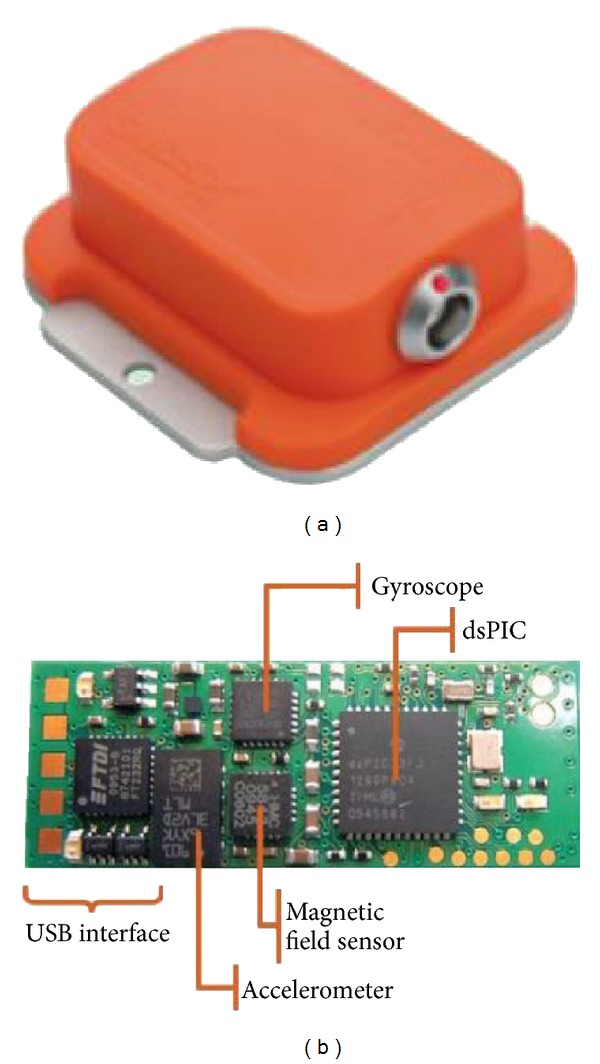
IMU sensor (a) and the prototype board (b).

 In our application, a sensor module, which is a strap down inertial measurement unit (IMU) is attached to the robot tool whose orientation (roll, pitch, and yaw) is to be determined.  Figure 3 shows an IMU of *Xsens* and its prototype board. The IMU sensor consists of one three-axis accelerometer, two two-axis gyroscopes, and one three-axis magnetometer.

We define three frames: body frame *x*_*b*_ *y*_*b*_ *z*_*b*_, sensor frame *x*_*s*_ *y*_*s*_ *z*_*s*_, and Earth-fixed frame *x*_*e*_ *y*_*e*_ *z*_*e*_. The sensor frame *x*_*s*_ *y*_*s*_ *z*_*s*_ corresponds to the axes of three orthogonally mounted accelerometers/magnetometers. Because the sensor is attached to the robot tool rigidly, the body frame *x*_*b*_ *y*_*b*_ *z*_*b*_ is assumed to coincide with the sensor frame *x*_*s*_ *y*_*s*_ *z*_*s*_. The Earth-fixed frame *x*_*e*_ *y*_*e*_ *z*_*e*_ is defined to follow the North-East-down (NED) convention, where *x*_*e*_ points North, *y*_*e*_ points East, and *z*_*e*_ points down. The IMU can measure the orientation (roll, pitch, and yaw) of itself. Define that the rotation *ϕ* about the *x*_*e*_-axis represents roll, the rotation *θ* about *y*_*e*_-axis represents pitch, and the rotation *ψ* about *z*_*e*_-axis represents yaw. According to Euler's theorem [33] on finite rotations, the conversion from Euler angles to quaternions is

(6) q=[q0q1q2q3]=[cos⁡(ϕ2)cos⁡(θ2)cos⁡(ψ2)+sin(ϕ2)sin(θ2)sin(ψ2)sin(ϕ2)cos⁡(θ2)cos⁡(ψ2)−cos⁡(ϕ2)sin(θ2)sin(ψ2)cos⁡(ϕ2)sin(θ2)cos⁡(ψ2)+sin(ϕ2)cos⁡(θ2)sin(ψ2)cos⁡(ϕ2)cos⁡(θ2)sin(ψ2)−sin(ϕ2)sin(θ2)cos⁡(ψ2)],

and the four Euler parameters are constrained as [34]

(7) $$\begin{array}{c} q_{0}^{2}+q_{1}^{2}+q_{2}^{2}+q_{3}^{2}=1, \end{array}$$

where *q*_0_ is the scalar part and (*q*_1_, *q*_2_, *q*_3_) is the vector part. So the direction cosine matrix *M*_*s*_^*e*^ from the sensor frame to the Earth-fixed frame can be represented as follows:

(8) $$\begin{array}{c} M_{s}^{e}=\left[\begin{array}{ccc} q_{0}^{2}+q_{1}^{2}-q_{2}^{2}-q_{3}^{2} & 2\left(q_{1}q_{2}-q_{0}q_{3}\right) & 2\left(q_{0}q_{2}+q_{1}q_{3}\right)\\ 2\left(q_{1}q_{2}+q_{0}q_{3}\right) & q_{0}^{2}-q_{1}^{2}+q_{2}^{2}-q_{3}^{2} & 2\left(q_{2}q_{3}-q_{0}q_{1}\right)\\ 2\left(q_{1}q_{3}-q_{0}q_{2}\right) & 2\left(q_{0}q_{1}+q_{2}q_{3}\right) & q_{0}^{2}-q_{1}^{2}-q_{2}^{2}-q_{3}^{2} \end{array}\right]. \end{array}$$

### 3.2. Quaternion KF

 Because both gyroscopes and magnetometer have white noise and random walk, we use Kalman Filter to estimate the state *x* of IMU from a set of noisy and incomplete measurements [35]. The Kalman Filter is a recursive stochastic technique and it estimates the state at time *k* from the state at time *k* − 1. The state-transition equation at time *k* is

(9) xk=Ak·xk−1+B·uk−1+wk−1,zk=H·xk+vk,

where *x*_*k*_ is the *n* × 1 state vector at time *k*, *A* is an *n* × *n* state-transition matrix, *B* is an *n* × *p* system input matrix, *u*_*k*−1_ is a *p* × 1 vector with deterministic input at time *k* − 1, *w*_*k*−1_ is an *n* × 1 process noise vector at time *k* − 1, *z*_*k*_ is an *m* × 1 measurements vector at time *k*, *H* is an *m* × *n* observation matrix, and *v*_*k*_ is an *m* × 1 measurement noise vector. In this paper, *n* = 7 and *m* = 6. The differential equation of the quaternion *q* with respect to time is

(10) $$\begin{array}{c} \left[\begin{array}{c} \frac{\partial q_{0}}{\partial t}\\ \frac{\partial q_{1}}{\partial t}\\ \frac{\partial q_{2}}{\partial t}\\ \frac{\partial q_{3}}{\partial t} \end{array}\right]=\left[\begin{array}{cccc} q_{0} & -q_{1} & -q_{2} & -q_{3}\\ q_{1} & q_{0} & -q_{3} & q_{2}\\ q_{2} & q_{3} & q_{0} & -q_{1}\\ q_{3} & -q_{2} & q_{1} & q_{0} \end{array}\right]\cdot \left[\begin{array}{c} 0\\ \frac{v_{x}}{2}\\ \frac{v_{y}}{2}\\ \frac{v_{z}}{2} \end{array}\right], \end{array}$$

where *v*_*x*_, *v*_*y*_, and *v*_*z*_ are the angular velocity components of IMU in *x*_*s*_-, *y*_*s*_-, and *z*_*s*_-axis. Since the state *x*_*k*_ includes the quaternion states and the angular velocities, *x*_*k*_ has the following form:

(11) $$\begin{array}{c} x_{k}=\left[\begin{array}{ccccccc} q_{0,k} & q_{1,k} & q_{2,k} & q_{3,k} & v_{x,k} & v_{y,k} & v_{z,k} \end{array}\right], \end{array}$$

where *q*_0,*k*_, *q*_1,*k*_, *q*_2,*k*_, *q*_3,*k*_, *v*_*x*,*k*_, *v*_*y*,*k*_, and *v*_*z*,*k*_ are the quaternion states and the angular velocities at time *k*. From (10), the state-transition matrix is

(12) $$\begin{array}{c} A_{k}=\left[\begin{array}{ccccccc} 1 & 0 & 0 & 0 & -q_{1,k}\cdot \frac{\Delta t}{2} & -q_{2,k}\cdot \frac{\Delta t}{2} & -q_{3,k}\cdot \frac{\Delta t}{2}\\ 0 & 1 & 0 & 0 & q_{0,k}\cdot \frac{\Delta t}{2} & q_{3,k}\cdot \frac{\Delta t}{2} & q_{2,k}\cdot \frac{\Delta t}{2}\\ 0 & 0 & 1 & 0 & q_{3,k}\cdot \frac{\Delta t}{2} & q_{0,k}\cdot \frac{\Delta t}{2} & -q_{1,k}\cdot \frac{\Delta t}{2}\\ 0 & 0 & 0 & 1 & -q_{2,k}\cdot \frac{\Delta t}{2} & q_{1,k}\cdot \frac{\Delta t}{2} & q_{0,k}\cdot \frac{\Delta t}{2}\\ 0 & 0 & 0 & 0 & 1 & 0 & 0\\ 0 & 0 & 0 & 0 & 0 & 1 & 0\\ 0 & 0 & 0 & 0 & 0 & 0 & 1 \end{array}\right], \end{array}$$

where Δ*t* is the sampling time. Let *B* = 0^*n*×*p*^ because there is no control inputs. We use angular velocities to estimate the quaternion states, so the process noise vector is

(13) $$\begin{array}{c} w_{k}={\left[\begin{array}{ccccccc} 0 & 0 & 0 & 0 & w_{x} & w_{y} & w_{z} \end{array}\right]}^{T}, \end{array}$$

where *w*_*x*_, *w*_*y*_, and *w*_*z*_ are the process noises of the angular velocity. Because we use calibrated gyroscopes to measure the angular velocities, the observation matrix *H* is

(14) $$\begin{array}{c} H=\left[\begin{array}{cc} 0^{3\times 4} & I^{3\times 3} \end{array}\right]. \end{array}$$

In order to satisfy (7), the determined quaternion *q*_*k*_ at time *k* should be normalized by

(15) qk=[q0,kMq1,kMq2,kMq3,kM],M=q0,k2+q1,k2+q2,k2+q3,k2.

## 4. Parameter Identification

Kinematic identification is the process that identifies the kinematic model parameters of a robot manipulator by a given set of robot tool pose measurements and the corresponding joint position readings. The objective of a kinematic identification algorithm is to minimize the difference between the computed and the measured poses [15].

Assuming that the number of measured pose is *m*, it can be stated that

(16) K^=K^N0=(K^(u1,v),K^(u2,v),…,K^(um,v))T,ΔT^=ΔT^N0=(ΔT^(u1,v),ΔT^(u2,v),…,ΔT^(um,v))T,

where **u**_*i*_ (*i* = 1, 2,…, *m*) is the vector of joint variables for the *i* measure pose.

All matrices or vectors in bold are functions of *m*. The objective of the kinematic identification is the computation for the parameter vector v* = *v*′ + Δ*v*, which is to minimize the discrepancy between the computed and the measured poses:

(17) $$\begin{array}{c} A\left(v^{*},u\right)=B\left(u\right) \end{array}$$

*A* is the function of pose of $\hat{T}$ and *B*(**u**) = (*B*(*u*_1_),  *B*(*u*_2_),…*B*(*u*_*m*_))^*T*^ is the measured function of joint variables **u**.

For each measurement pose *B*(*u*_*i*_), it concludes orientation measurement *R*_*i*_ ∈ *ℜ* ^3×3^ and position measurement *P*_*i*_ ∈ *ℜ* ^3×1^, and

(18) $$\begin{array}{c} B\left(u_{i}\right)=\left[\begin{array}{cc} R_{i} & P_{i}\\ 0 & 1 \end{array}\right],\;u_{i}\in {ℜ}^{4\times 4}. \end{array}$$

If the measurement system can provide orientation measurement and position measurement, each pose can formulate six measurement equations. If only orientation measurement can be provided by the measurement system, each pose measurement can just formulate three measurement equations. In this paper, only orientation obtained from IMU is used to calibration the kinematic parameters. From(17),

(19) $$\begin{array}{c} A\left(v^{*},u\right)=B\left(u\right)=A\left(v,u\right)+C\left(\Delta v,u\right), \end{array}$$

where *C* is the discrepancy function of the orientation components of $\Delta \hat{T}$. Introducing the Jacobian matrix,

(20) $$\begin{array}{c} C\left(\Delta v,u\right)=J\cdot \Delta v, \end{array}$$

and then

(21) $$\begin{array}{c} C\left(\Delta v,u\right)=B\left(u\right)-A\left(v,u\right), \end{array}$$

when using

(22) $$\begin{array}{c} b=B\left(u\right)-A\left(v,u\right)\in {ℜ}^{4\times 4\times m}, \end{array}$$

(23) $$\begin{array}{c} x=\Delta v\in {ℜ}^{4\times 4\times m}. \end{array}$$

Equation (20) can be rewritten:

(24) $$\begin{array}{c} J\cdot x=b. \end{array}$$

## 5. Estimating Errors Using Extended Kalman Filter

Initially, the orientations of the tool are measured from the IMU. Since uncertainty exists in the measurement, Extended Kalman Filter (EKF) is used as an optimization algorithm and the Jacobian matrices are used to estimate the kinematic errors of DH parameters by the measured orientation values [5].

Since there are four parameters for *N* revolute joints and four parameters for the transformation from the IMU to the tool, the number of total parameters to be considered is 4(*N* + 1). So the predicted state $\hat{x}$ is 4(*N* + 1) of the DH parameters in the prediction step of the EKF. The covariance matrix of the predicted state *P* is

(25) x^k+1|k=x^k|k,Pk+1|k=Pk|k+Qk,

where *Q*_*k*_ is the covariance matrix of the system noise at the *k*th iteration. 

In the observation step of the EKF, Jacobian matrix *J* (20), measurement residual $\tilde{y}$, and residual covariance *S* are calculated as follows:

(26) Jk+1=∂T(x)∂x|x^k+1|k,y~k+1=mk+1−T(x~k+1|k),Sk+1=Jk+1Pk+1Jk+1T+Rk+1,

where *m*_*k*_ and *R*_*k*_ are the measured orientation value and the covariance matrix of measurement noise at the *k*th iteration. *k* + 1 | *k* means a prior estimate, and *k* + 1 | *k* + 1 means a posteriori estimate.

In the update step, the state covariance matrix is updated by an optimal Kalman gain *K*:

(27) Kk+1=Pk+1|kJk+1TSk+1−1,x^k+1|k+1=x^k+1|k+Kk+1y~k+1,Pk+1|k+1=(I−Kk+1Jk+1)Pk+1|k,

where *I* is the identity matrix. The norm values of the state vector are calculated for every iteration once the updating procedure is completed. Note that if *Q* and *R* are set to zero, then EKF simply reduces to the Newton-Raphson method.

## 6. Experimental Results

### 6.1. Experiments Environment

 To verify the above method, a GOOGOL GRB3016 robot model was used in this experiment. In the experiment, the robot could self-calibrate online in the working status. There were four steps in the self-calibration procedure.

1. Data collection: the orientation of the IMU and the corresponding joint angles were captured with the robot tool moving in different pose.
2. Manipulator orientation estimation: the orientations of the manipulator were estimated via the KFs from the obtained data.
3. Kinematic parameters identification: the manipulator kinematic parameters were identified from the estimated orientations and the joint data.
4. Calibration accuracy assessment: 3D pose errors were used to verify the calibration accuracy by inserting a peg into the holes (Figure 5).

**Figure 4:**
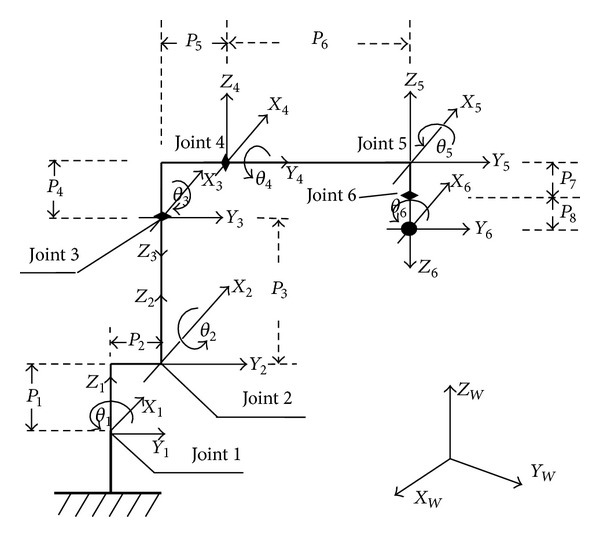
Skeleton of the GOOGOL GRB3016 robot with coordinate frames in the zero position.

Table 1 lists the nominal robot link parameters of the robot, which were chosen as the initial conditions for the above kinematic identification algorithm, and Figure 4 shows the skeleton of the GOOGOL GRB3016 robot with all coordinate frames and geometric features. As expected, the increase in the noise intensity will lead to the increase in the calibration errors.

 From Table 1 note that a GOOGOL GRB3016 robot with 6 DOF needs 24 geometric parameters to be modeled. From (18), each 3D robot orientation provides 3 model equations. So a unique computation of the 24 parameters needs 8 pose measurements at least. And more pose measurements will decrease the calibration errors. But limited by the measurement accuracy (affected by the noise), the calibration errors intend to be stable as the pose measurements increase.

**Figure 5:**
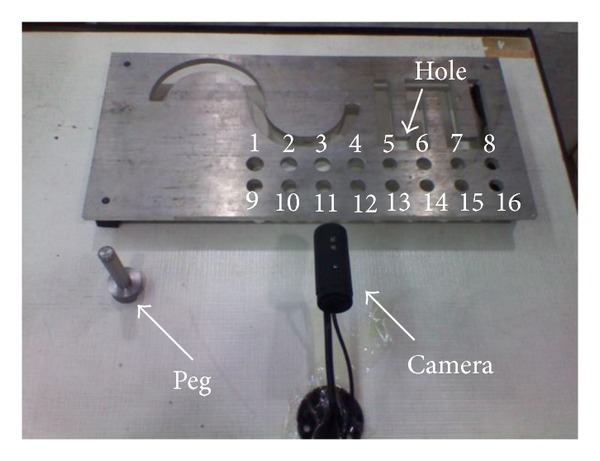
Steel plate and errors measurement system.

 In order to validate the proposed IMU-based robot calibration, we have performed a number of peg-into-hole experiments. In our experiments, two calibration methods were used to carry out the experiments of peg-into-hole. Our method was initially compared with a standard vision-based robot calibration of the Meng and Zhuang [16]. There were 16 holes in the steel plate (Figure 5) and 16 tests of peg-into-hole were carried out in each experiment. The peg was the cylinder with 7.5 mm in radius and 150 mm in length. The radius of the hole was 8 mm. The size of the steel plate was 300 mm × 500 mm. In the step of our method, an IMU (Xsens MTi-100 IMU) was rigidly attached to the robot tool flange to measure the pose of the robot tool. The static accuracy of roll and pitch was 0.02 deg and that of yaw was 0.05 deg. The dynamic accuracy of roll and pitch was 0.05 deg and that of yaw was 0.1 deg. The noise density of gyroscopes was  $0.01\;\deg/\text{s}/\sqrt{\text{Hz}}$ and that of the magnetometer was  $200\;\mu \text{G}\;\sqrt{\text{Hz}}$ . The IMU measurements were received at 100 Hz. Since the robot will be stopped after it executes a command, in order to improve the accuracy, the system collects the static IMU data after the robot stops. The coordinates of the center of the hole with respect to the base coordinate system were known. In the step of method [16], a camera, with 1280(H) × 960(V) picture elements and 22 frame/s frequency, was mounted on the robot tool to capture the RGB images of the chessboard. A chessboard pattern, the position of which was unknown in the reference frame, was placed on the platform. The distances, measured by a caliper, between two adjacent corners of the square were known. Note that the robot has different kinematic parameter errors in the different position. In order to improve the accuracy, the system made a robot calibration before inserting the peg into each hole. 

**Figure 6:**
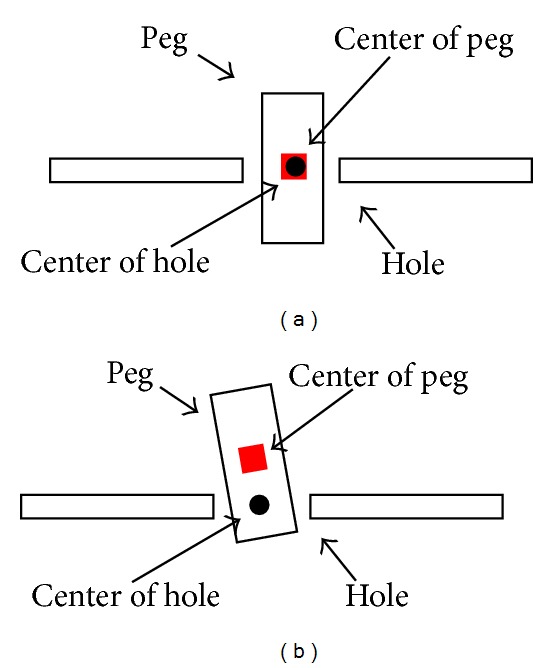
Definition of 3D errors.

 To evaluate the calibration accuracy, 3D position errors between the peg and the hole were proposed. A calibrated camera with 1280(H) × 960(V) picture elements was used to measure the 3D position errors (Figure 5). In the initiation of the system, the camera measured the coordinates of the center of the hole with respect to the camera coordinate system. After the peg was inserted into a hole, the camera can measure the depth and the direction of insertion by detecting the edge of the peg. And then the system could calculate the center of the peg with respect to the camera coordinate system. Let (*x*_*o*_, *y*_*o*_, *z*_*o*_) and (*x*_*t*_, *y*_*t*_, *z*_*t*_) be the centers of the peg and the hole, respectively (Figure 6). Then the 3D position error *E* can be written:

(28) $$\begin{array}{c} E=\sqrt{{\left(x_{o}-x_{t}\right)}^{2}+{\left(y_{o}-y_{t}\right)}^{2}+{\left(z_{o}-z_{t}\right)}^{2}.} \end{array}$$

The mean absolute error in position for *N* peg-into-hole tests was

(29) $$\begin{array}{c} E_{m}=\overset{N}{\underset{k=1}{\sum }}\frac{E_{k}}{N}, \end{array}$$

where *E*_*k*_ is the 3D position error for the *k*th hole.

### 6.2. Result Analysis

**Figure 7:**
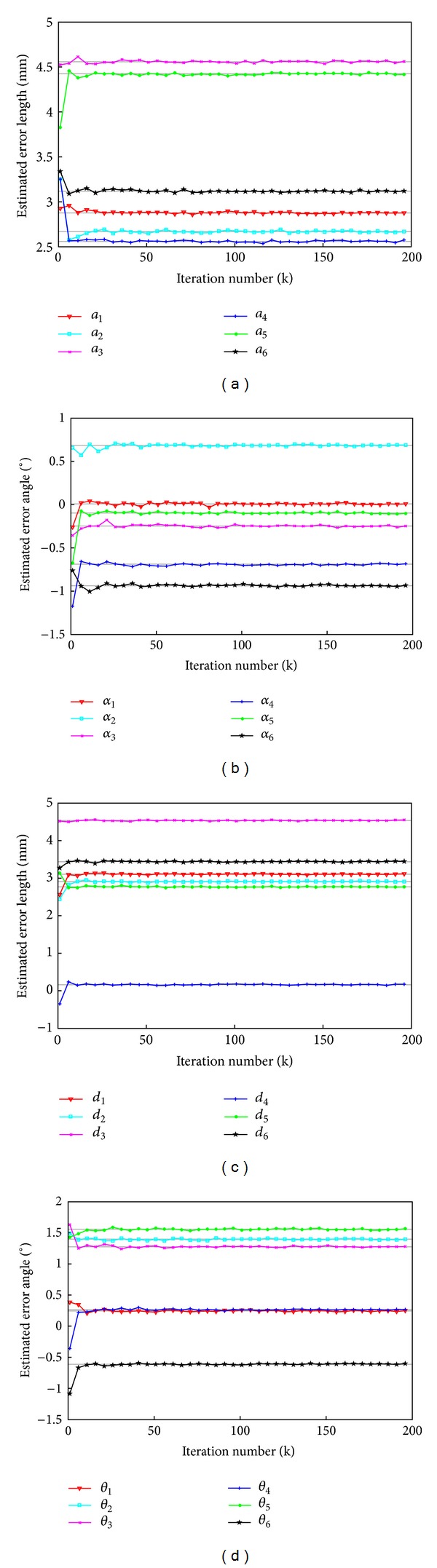
Estimated *D*-*H* parameter errors with EKF.

Following the initialization step, the system performs a series of tests for peg-into-hole. The KF processes the IMU measurements and concurrently estimates the state vector. For EKF, the number of maximum iteration was set as 2000. *Q* and *R* were set as 1.0 × 10^−3^ × *I*_3×3_. When the state vector was less than 1.0 × 10^−5^, the iteration terminated. The proposed algorithm was evaluated by estimating the orientation from the IMU and the theoretical orientation obtained by using (3).  Figure 7 shows the estimated DH parameter error values of the robot tool. As shown in Figure 7, the EKF algorithm quickly converged to the stable error value from about the fifteenth iteration.

Table 2 shows the estimated parameter errors when the calibration sets were used in EKF fro 200 iterations. Since the EKF algorithm quickly converged to the stable error value from about the fifteenth iteration, The iteration should be set as 15 so that the calibration could be carried out in real time.

In the experiments of peg-into-hole, the iteration was set as 15 to estimate the parameter errors. The state-estimate errors with 15 pose measurements are shown in Table 3. As evident from Table 3, even with the noise error for the IMU measurements, the algorithm is still able to attain very accurate estimates of the calibration parameters. Table 3 shows the accuracy result of method [16] too. By comparing the results of Table 3, the calibration parameters were more accurate in our method.

**Figure 8:**
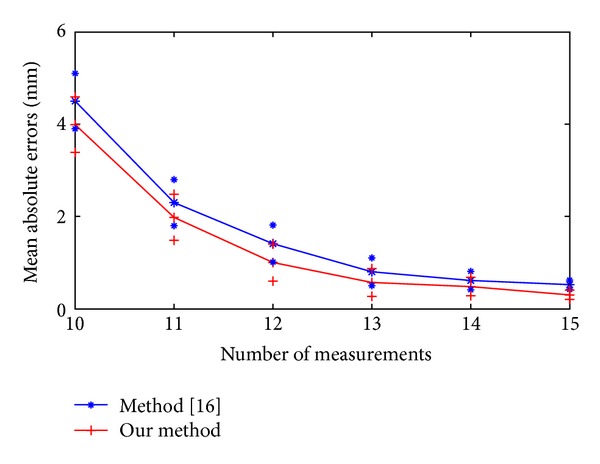
Mean absolute errors in different number of measurements.

In our method, a unique computation of the 24 kinematic parameters needs 8 pose measurements. In method [16], it needs 4 pose measurements at least. We compare two methods from 10 pose measurements to 15 pose measurements. The results presented in Figure 8 show that with more pose measurements, the parameter error decreases gradually. The mean absolute estimation errors from 10 pose measurements to 15 pose measurements in method [16] were 4.5 mm, 2.3 mm, 1.41 mm, 1.3 mm, 0.61 mm, and 0.52 mm with standard deviations (SDs) of 0.4 mm, 0.38 mm, 0.35 mm, 0.28 mm, 0.09 mm, and 0.09 mm. Compared with method [16], the mean absolute errors of our method drop about 0.51 mm, 0.32 mm, 0.41 mm, 0.23 mm, 0.13 mm, and 0.22 mm. The estimation errors trend slows down after two times of the minimum number of poses measurements.

**Figure 9:**
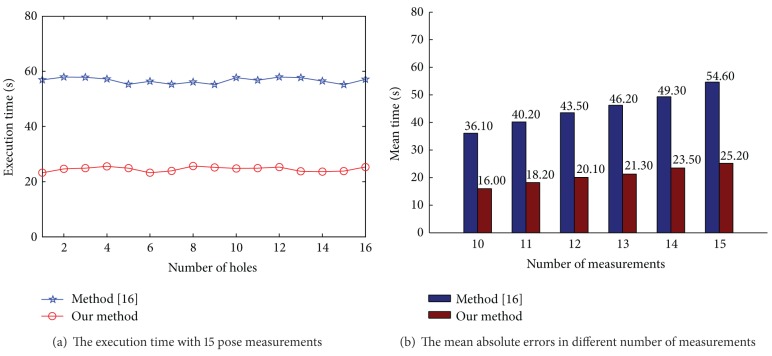
The comparison in execution time.

Compared with method [16], the great advantage of our method is that the system does not need to make a more motion to take the photo. After the robot executed a command, the robot would stop and the system concurrently obtained the static measurement data from the IMU.  Figure 9(a) shows the execution time of two methods with 15 pose measurements. In method [16], the average time of taking a photo was 3 s. In our method, the station time of robot was about 1 s. The execution time of peg-into-hole is about 8 s. The time of parameter identification was 0.8 s.  Figure 9(b) shows the comparison of execution time with different pose measurements. The time in method [16] is more than two times that in our method.

## 7. Conclusions

An IMU-based online autonomous calibration for serial robot has been proposed in this paper. In this approach, the IMU is rigidly attached to the robot tool to estimate the robot pose automatically during the working time. An efficient approach which incorporates Factored Quaternion Algorithm (FQA), Kalman Filter (KF), and Extended Kalman Filter (EKF) to estimate the orientation of the IMU is presented in this paper. After the robot poses are estimated, the kinematics identification can be carried out. Finally, the robot kinematic parameters can be corrected from the identification results in real time. The whole procedure of the robot calibration is automatic and without any manual intervention. The results of the experiments show the good accuracy, convenience, and effectiveness of the presented approach.

Compared with the existing expensive and complex approach, the method this paper proposed is easier to use and setup. Compared to the existing vision-based self-calibration method, the proposed method can conduct the calibration more accurately and with less execution time. In the future work, we will research the approach which can accurately estimate the robot pose without stopping the robot. With the dynamic pose measurements, the robot calibration will be more efficient.

## Figures and Tables

**Table 1:** The nominal link parameters in DH model for the GOOGOL GRB3016 robot.

Joints	DH			
	*a*	*α*	d	*θ*
1	150	−*π*/2	250	0
2	570	−*π*	0	−*π*/2
3	150	*π*/2	0	0
4	0	−*π*/2	650	0
5	0	−*π*/2	0	−*π*/2
6	0	0	−200	0

**Table 2:** Estimated parameter errors of 6 DOF robot.

Error	Joint					
	1	2	3	4	5	6
Δ*a* (mm)	2.8759	2.6679	4.5582	2.5580	4.4248	3.1172
Δ*α* (°)	0.0088	0.6855	−0.2482	−0.6892	−0.0974	−0.9373
Δ*d* (mm)	3.1049	2.9095	4.5347	0.1594	2.7675	3.4382
Δ*θ* (°)	0.2441	1.3982	1.2752	0.2644	1.5575	−0.6128

**Table 3:** The 3D errors with 15 pose measurements.

Hole number	Method [16] (mm)	Our method (mm)
1	0.45	0.40
2	0.33	0.30
3	0.51	0.29
4	0.52	0.21
5	0.38	0.32
6	0.61	0.16
7	0.61	0.37
8	0.49	0.46
9	0.53	0.23
10	0.51	0.38
11	0.48	0.42
12	0.57	0.14
13	0.44	0.15
14	0.71	0.35
15	0.48	0.26
16	0.51	0.36
